# Modelling urban vibrancy with mobile phone and *OpenStreetMap* data

**DOI:** 10.1371/journal.pone.0252015

**Published:** 2021-06-02

**Authors:** Federico Botta, Mario Gutiérrez-Roig

**Affiliations:** 1 Department of Computer Science, University of Exeter, Exeter, United Kingdom; 2 Department of Mathematical Sciences, University of Essex, Colchester, United Kingdom; King Abdulaziz University, SAUDI ARABIA

## Abstract

The concept of urban vibrancy has become increasingly important in the study of cities. A vibrant urban environment is an area of a city with high levels of human activity and interactions. Traditionally, studying our cities and what makes them vibrant has been very difficult, due to challenges in data collection on urban environments and people’s location and interactions. Here, we rely on novel sources of data to investigate how different features of our cities may relate to urban vibrancy. In particular, we explore whether there are any differences in which urban features make an environment vibrant for different age groups. We perform this quantitative analysis by extracting urban features from *OpenStreetMap* and the Italian census, and using them in spatial models to describe urban vibrancy. Our analysis shows a strong relationship between urban features and urban vibrancy, and particularly highlights the importance of *third places*, which are urban places offering opportunities for social interactions. Our findings provide evidence that a combination of mobile phone data with crowdsourced urban features can be used to better understand urban vibrancy.

## Introduction

Recent years have witnessed an increased urbanisation of the environment in which people live, with more than half of the world’s population living in urban areas [1]. Cities are developing at an unprecedented pace and are becoming hubs of innovation, social interactions, economic growth and globalisation [2, 3]. However, the large diversity of cultures and people living in metropolitan areas also poses challenges in terms of economic inequality and criminality [4, 5].

Traditionally, quantitative studies of urban environments have been difficult due to the lack of suitable data to measure urban vibrancy, as well as to quantify urban environments. However, the recent surge in large data sets derived from our interactions with large technological systems, such as the Internet, offers novel opportunities in this area, which has become known as urban analytics or urban computing [6]. In particular, our regular use of mobile phones and social media platforms generates a vast amount of data on our behaviour and our mobility [7, 8]. High resolution data on urban environments is now also widely available at a large scale thanks to open collaborative projects, such as *OpenStreetMap* [9].

Recent studies have used these new forms of data to measure our society [10–15], our cities [16, 17] and how people perceive them [18–23]. Deep learning tools have even allowed us to extract information about how cities look [24, 25].

*Urban vibrancy*, also referred to as *urban vitality*, is a central notion in the study of cities. Despite it being a multifaceted concept, broadly speaking it refers to a measure of a dynamic, positive and energetic activity in an urban environment. For instance, social or commercial activities can foster the vibrancy of a neighbourhood. Urban sociologists and designers have studied this and related concepts for decades [26, 27] with different approaches and hypothesis. Jane Jacobs’ book *The Death and Life of Great American Cities* [26] has had an enormous influence in the development of theories of urban design, and is still greatly influencing research in urban systems [28–30]. In her book, Jacobs discusses a wide range of aspects related to urban environments, and provides several interesting perspectives on how urban planners can encourage a vibrant urban life. The presence of dense built environments, with roads and pedestrian paths that favour spontaneous interactions, is suggested to be a key component of what may make an environment vibrant. In Jacobs’ view, built environments need to be dense enough that they can attract people, both in residential and nonresidential neighbourhoods. However, density alone may not be enough, and Jacobs argues for the presence of a diverse range of buildings, both in terms of their structure, but crucially also in their age. Buildings of a diverse age will encourage a wide range of different activities. Additionally, the presence of older buildings in modern neighbourhoods shows creativity in adapting to the changes of time. It can also foster the presence of people of different socioeconomic backgrounds [31, 32]. Another important aspect is the presence of places that favour social cohesion and interactions, such as bars, cinemas and shopping centres. The notion of *third places* was introduced to refer precisely to these places which can foster social activities which do not take place at home or at work, respectively *first* and *second places* [33, 34]. The importance of *third places* is linked to the opportunities that they offer “to escape” from the usual first two places of life, where most of our time is spent. They allow us to develop social relationships which can lead to a sense of wellbeing and social accomplishment. Additionally, *third places* encourage new social encounters since they are populated by “a shifting diversity of inhabitants […]. As a result, an aura of the unexpected surrounds each visit to a third place” [33].

Measuring urban vibrancy has traditionally been challenging, but the availability of many new data sets has allowed researchers to build proxies for it from social media check-ins, Oyster card data, bank transactions and public WiFi usage [35–38]. We can use this new data sets as a measure for the vibrancy of an area because they have been shown to provide good estimates of how many people are present in that location [14, 15]. In the last few years, researchers have used these large data sets to gain insight into several aspects of what urban features make an environment vibrant [38–41], as well as what the relationship is between a vibrant area and its safety [42]. Mobility is an important aspect of urban vibrancy, and smart phone apps can be used to better track and understand our daily movements [43]. Finally, thorough tests of Jane Jacobs’ conditions have been performed in a range of cities [28, 29, 44, 45] and have supported her theories of urban vibrancy.

Our analysis contributes to the literature on urban vibrancy in several ways. First, we build on existing studies, which have investigated the relationship between a range of urban features and vibrancy in urban environments, by investigating whether the same relationships hold even when measuring urban vibrancy by age groups of people. To do so, we use a large data set on the presence of mobile phone users from different age groups in seven large Italian cities, and we combine this information with data from the Italian census and *OpenStreetMap*, an open-source collaborative mapping project. As discussed above, existing studies have shown how different urban features are related to urban vibrancy, and have tested urban theories by using proxy measures for measuring vibrant neighbourhoods. However, we hypothesise that what makes an urban environment vibrant may be different for people of different ages. For instance, a neighbourhood with many bars and clubs may be vibrant for younger people, but would not be for older age groups. When aggregating vibrancy across all age groups, these differences may vanish or be significantly reduced. In our analysis, we aim to investigate whether such differences exist within our data set, and to provide a better understanding of the diverse nature of urban vibrancy.

Our first analysis will consider all cities together. However, our second novel contribution is a city-level analysis where we explicitly model the spatial dependencies in our data to investigate whether any effect found in the first analysis could be due to spatial correlations that have not been accounted for.

Finally, we focus more specifically on the notion of *third places*. *Third places* capture social interactions that happen outside of home and work, and they offer unique opportunities for community building [33, 34]. Understanding their relationship to urban vibrancy is key in designing neighbourhoods which can foster a positive environment for people living there.

## Data

### Mobile phone data

**Fig 1:**
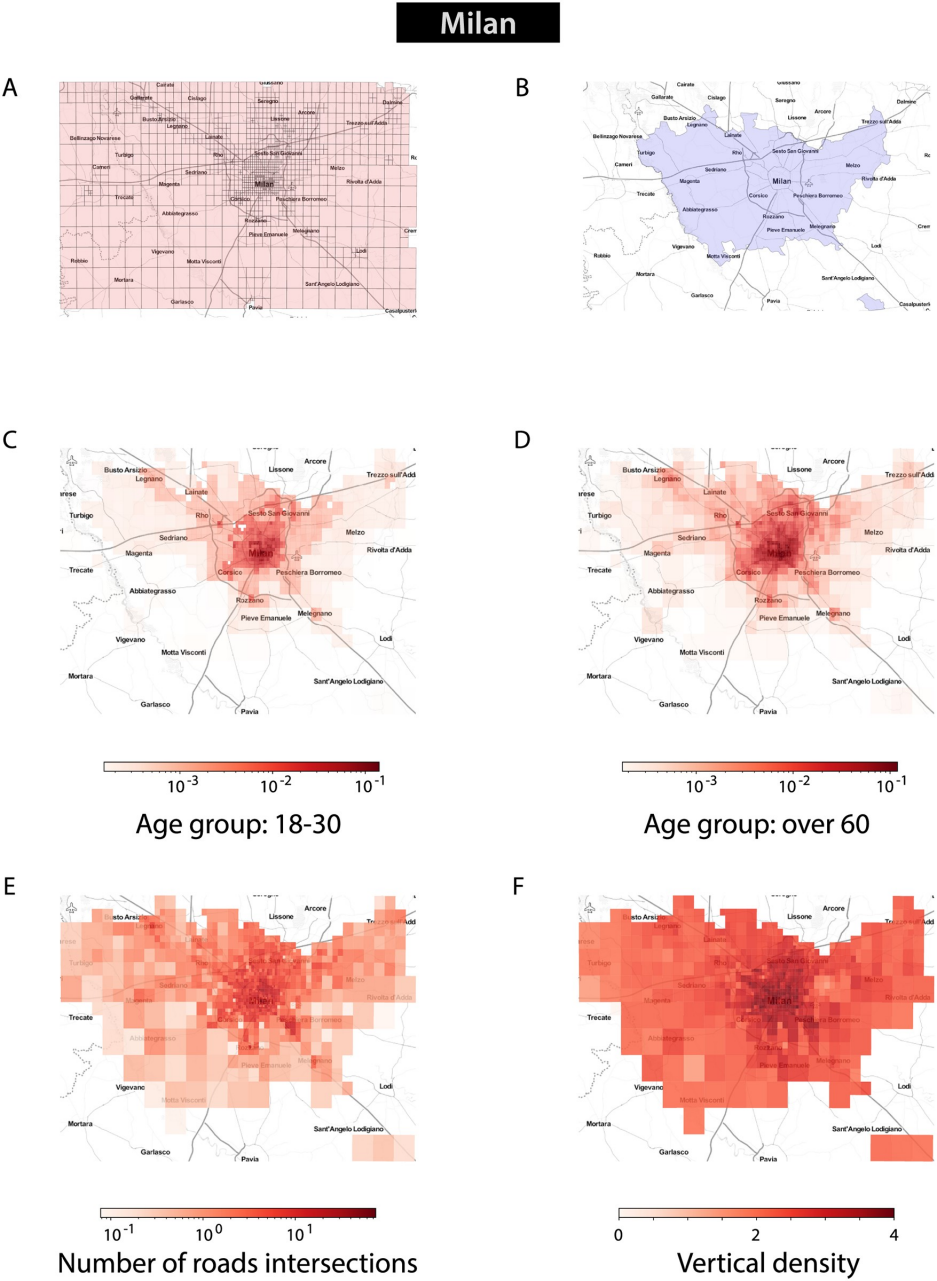
Presence of mobile phone users and density of urban features in Milan. We retrieve data on the number of mobile phone users in the city of Milan, together with their age group, as derived from *Telecom Italia* data between March and April 2015. (A) Mobile phone users are provided for cells in a discrete grid superimposed on the city. We depict here all the cells which are included in the dataset for the metropolitan city of Milan. (B) Our analysis focuses on urban environments in densely populated areas. We present here the geographical boundaries of the administrative unit of the metropolitan city of Milan, which we use to select those cells in the data set which are part of the large urban area of Milan. (C, D) We depict here the average density of users for two different age groups across the area of analysis. Visual inspection suggests that the overall pattern is consistent across the two groups (E, F) We compare the presence of people across the city with the underlying urban structure of the different cells. To quantify the urban environment, we consider data derived from *OpenStreetMap* (OSM) and the Italian census. Here, we present (E) the number of road intersections extracted from OSM, and (F) the vertical density of buildings. All maps in this Figure are oriented North.

We retrieve data on mobile phone activity recorded in seven Italian cities: *Milan, Rome, Turin, Naples, Palermo, Bari and Venice*. The data set covers a period of two months from March 2015 until the end of April 2015, and was released by *Telecom Italia*, an Italian mobile provider, as part of their *Big Data Challenge 2015* [46]. In their challenge, *Telecom Italia* only released the data set for the two month period used in our analysis, so our analysis is restricted to this time period. Mobile phone activity measurements are provided at 15 minute granularity, for cells in a discrete grid superimposed on the area of each city. The cells were designed by *Telecom Italia* and are not regular in size. Their dimension changes in relation to the dimension of the underlying mobile cells, but the latter are not provided by *Telecom Italia*. Fig 1A depicts the spatial grid over the city of Milan and its surroundings.

The mobile phone activity data set contains the number of *Telecom Italia* users who were in each cell at a 15 minute granularity. The number of users in each cell has been calculated by *Telecom Italia* from *Call Detail Records* generated by outgoing calls taking place on their cellular network. We refer to this data set as the presence of *Telecom Italia* users. If a given cell and time interval resulted in fewer than three users, it is discounted for privacy concerns. Additionally, all values are rescaled by the same unknown factor in order to further preserve privacy.

The presence of *Telecom Italia* users is also further divided into the following age groups: *less than 18*, *[18–30]*, *[31–40]*, *[41–50]*, *[51–60]*, and *over 60*. For each cell and for each time interval, the data set contains the number of *Telecom Italia* users in each of these age groups. Fig 1C and 1D depict the average density of users for two different age groups in Milan. Initial visual inspection suggests that the presence of users across Milan is similar for the different age groups. We use the mobile phone data set as a proxy for urban vibrancy in the cities under analysis, in line with other studies which have used new forms of data as a proxy for urban vibrancy [28, 35–38]. In this analysis, we aggregate the presence data over the whole time period to get a single measure of urban vibrancy in each cell.

### Metropolitan cities

In our analysis, we are interested in the relationship between urban vibrancy and geographical features of urban environments. To ensure that we focus our study on densely populated urban areas, we take into account the administrative units of Italy known as metropolitan cities. Metropolitan cities are an administrative division introduced in 2014 and they include a large populated city and the surrounding towns which are closely related to it, both economically and culturally.

We retrieved data on the boundaries of the metropolitan cities under analysis from the website of the Italian office for national statistics *ISTAT* [47]. Fig 1B depicts the boundary of the metropolitan city of Milan. Our analysis only considers cells which appear both in the spatial grid provided by *Telecom Italia* as well as within the boundary of the corresponding metropolitan city. Table 1 presents the population of the seven metropolitan cities under consideration, and the number of mobile phone cells which are included in each of them.

**Table 1:** Metropolitan cities. Our analysis focuses on seven metropolitan cities across Italy. Here, we report the number of spatial cells of the mobile phone network and the population (in thousands) of each of these cities split across 6 age groups. Population data is retrieved from the 2011 Italian census and comprises all the census sections within the phone cells considered for each city. It is important to highlight that in each cell of the network there can be several mobile phone users, thus we cannot estimate the fraction of the census population included in our data set. Note that the age groups provided by the Italian census do not perfectly match those of the *Telecom Italia* dataset.

City	N° cells	Pop. (in 1,000)	<20	20–29	30–39	40–49	50–59	>60
Milan	921	5,454	992	513	791	929	711	1,518
Rome	843	6,313	1,181	634	918	1,083	848	1,649
Turin	535	3,294	575	311	454	533	444	977
Naples	355	4,826	1,165	646	700	744	603	968
Venice	248	1,412	252	131	197	244	194	394
Palermo	165	1,709	370	217	240	257	223	402
Bari	143	1,555	318	190	225	245	205	372

### Census data

Information about the population, and where people live, is collected by countries through census campaigns. Italy carried out its last census in 2011, and we retrieve the corresponding data from the *ISTAT* website. The lowest basic territorial unit is the census section (*sezione di censimento*), which cannot be bigger than a local council and contains around 250 households. The census provides a large amount of information for each census section, but for the purpose of this study we are interested in two main features. Firstly, we extract the number of people registered as living in each census section divided by age groups: less than 20 years old, 20–29, 30–39, 40–49, 50–59, and 60 or more years old. These groups closely match the age groups of the mobile phone data. Secondly, we retrieve data on the structure of buildings in each census section. More precisely, we retrieve data on the number of buildings, their age and the number of floors. We use this information as geographical features in our analysis, along with further features described in the next subsection. The ISTAT website provides shapefiles for every Italian region. From these, we retrieve the geographical boundaries for the census sections within the seven metropolitan cities under analysis. In order to be able to merge the mobile phone data and the data from the census we join them using weighted area spatial aggregation. This method consists of computing the weighted average of all the census polygons that fall within the mobile phone grid polygons. Note that, on average, cells in the mobile phone grid are much bigger than census sections. The weight of each census section is proportional to the intersecting area in the cell polygon.

### *OpenStreetMap* data

Measuring geographical features of urban environments is a challenging task. Cities are spread over a large spatial area, with a variety of different types of spaces, buildings, roads, and so on. A systematic way of capturing this information has only been recently possible thanks to the availability of open data collected via collaborative and crowdsourcing projects. In particular, *OpenStreetMap* (OSM) is a collaborative project providing free map data used by millions of people across the globe. Registered users can add and edit features to the map in a similar way to Wikipedia. Here, we retrieved OSM data for Italy via the *Geofabrik Downloads* website [48], which provides raw OSM data for countries worldwide. Data was retrieved on March 20th 2019. It is important to highlight that OSM data was retrieved in a different time period compared to the mobile phone data. Whilst this temporal difference may give rise to some inconsistencies in the analysis, we also note that physical features of urban environments are unlikely to be drastically different between 2015 and 2019 for the cities under analysis.

## Data preparation

Before starting our analysis, we perform an initial processing step on the data. Data from the census and from OSM are available at a very granular spatial resolution. However, data from mobile phone activity is available for geographical cells which tend to be larger than the census sections. Therefore, we first aggregate the census and OSM data at the same spatial level of the mobile phone activity data.

All data, with the exception of the indices of diversity defined below, is normalised by the area of the spatial cell at which the data is aggregated. Therefore, whenever we refer to the presence of mobile phone users or to features of the urban environment, in reality this is the density of such variables.

### Diversity index

The presence of a diverse set of urban features may encourage a more vibrant environment thanks to the greater number of opportunities that those features may offer. Throughout our analysis, we will measure the diversity of a set of urban features using Shannon’s definition of diversity, which is based on the notion of entropy [49]. The diversity index *δ* is calculated as follows:

$$\begin{array}{c} \delta =-\sum_{i=1}^{n}p_{i}\ln p_{i} \end{array}$$

where *n* is the total number of categories in the considered feature, and *p*_*i*_ is the frequency of category *i*. For instance, *n* could be the total number of types of *Points of Interest*, and *p*_*i*_ the frequency of each type.

### Points of interest

From the OSM dataset, we extract data on *Points of Interest* (POIs) and *Points of Worship* (POWs). POIs and POWs include many types of places, such as cafes, restaurants, churches, and monuments. We aggregate together all POIs and POWs in each spatial cell, and from now on we refer to this simply as POIs. We use the diversity index *δ* defined above to measure the diversity of POIs in each cell, and we refer to this quantity as POIs *δ*. A larger POIs *δ* indicates the presence of a more diverse set of POIs in the OSM dataset.

Additionally, we introduce here the concept of *third places*, in line with the literature [33, 50]. *Third places* are places which are neither the place where a person lives (first place) nor their work place (second place), and they are a subset of the POIs and POWs which are available on OSM. They tend to represent places where people can carry out activities which are more social than others, provide opportunities for social interactions and community building. A four category classification for *third places* is commonly used [50]: *eating, drinking and talking* (such as cafes, bars, restaurants), *organized activities* (such as places of worship and clubs), *outdoor* (such as parks), and *commercial venues* (such as shopping centres). From the list of 289 POIs derived from OSM data for the cities under analysis, we manually label those which are *third places* to assign them to one of the four categories of *third places* whenever possible. We calculate the total number of *third places* in each cell falling in each category. We also calculate the total number of *third places* in each cell, as well as a measure of their diversity, which we call *third places* *δ*. In our analysis, we consider both POIs and *third places*, since they are related yet different measures. It is also important to highlight that both POIs and *third places* are uniquely derived only from OSM data, and that mobile phone data have not been used in this step.

### Roads

It is perhaps rather intuitive that the structure of the roads network can play a role in the life of an urban environment. Jacobs suggests that it is particularly the availability of road intersections which may encourage spontaneous social activity and support urban vibrancy [26]. For each spatial cell in our dataset, we retrieve all road segments from OSM and calculate the pairwise number of intersections between all roads that fall within the given cell.

### Buildings

From the census data, we extract the total number of buildings in each census section, and aggregate that to the same spatial level as the mobile phone data. We consider the number of buildings since a high urban density may foster vibrancy and a larger concentration of vibrant activities. However, in modern urban environments vertical development, i.e. the height of buildings, may also contribute to the density of a neighbourhood, as has been investigated in [28, 29, 35]. Therefore, from the census data we also construct a measure, analogous to that used in [28], of vertical density as follows:

$$\begin{array}{c} \text{vertical}\;{\text{density}}_{i}=\frac{\sum_{h}b_{h,i}f_{h}}{\sum_{h}b_{h,i}} \end{array}$$

where *b*_*h*,*i*_ is the number of buildings in height category *h* in cell *i*, and *f*_*h*_ is the number of floors in the same height category *h*. In the 2011 Italian census data, the possible height categories are: 1 floor, 2 floors, 3 floors, and 4 floors or more. The age of buildings has also been suggested to have a role in making an environment vibrant [26]. Jacobs particularly highlights how the coexistence of old and new buildings may be crucial in fostering the presence of enterprises, businesses, and people. The Italian census has information on the age of buildings divided in different age categories: buildings older than 1919; buildings built between 1919 and 1945; buildings built between 1946 and 1960; buildings built between 1961 and 1970; buildings built between 1971 and 1980; buildings built between 1981 and 1990; buildings built between 1991 and 2000; buildings built between 2001 and 2005; and finally buildings built from 2005 until the census date. Following [28], we calculate the average age of buildings in cell *i* as follows:

$$\begin{array}{c} {\text{Buildings}}^{\prime }\;{\text{ages}}_{i}=\frac{\sum_{a}b_{a,i}age_{b}}{\sum_{a}b_{a}} \end{array}$$

where *b*_*a*,*i*_ is the number of buildings in age category *a* in cell *i*, and *age*_*b*_ is:

$$\begin{array}{c} age_{b}=\frac{2011-start_{b}+2011-end_{b}}{2} \end{array}$$

and *start*_*b*_ and *end*_*b*_ being, respectively, the start and end date of each of the age categories defined above. Finally, we consider the diversity in the buildings’ ages in cell *i*, and we refer to it as Buildings′ ages *δ*.

## Methods

Our analysis focuses on the relationship between features of urban environments and a proxy measure of urban vibrancy in Italian cities. To investigate this relationship, we first carry out a non-parametric analysis using Kendall’s rank correlation coefficient between each urban feature and our proxy of urban vibrancy for different age groups. To correct for multiple hypothesis testing and control the expected proportion of false positives, we adjust all of the p-values in our correlation analysis using *false discovery rate (fdr)* correction [51].

The second step of our analysis focuses on spatial modelling of the data. The use of spatial models is crucial when dealing with data for which the location is not only available, but also important [52, 53]. The types of spatial models we consider in our analysis are spatial lagged models and spatial error models [54, 55]. These are standard regression models in which the spatial dependency is explicitly included, albeit in different ways. A spatially lagged model incorporates the spatial dependency directly in the model by including a spatially lagged variable amongst the predictor variables, whereas a spatial error models incorporates spatial effects in the error term of the regression equation. The use of Lagrange Multiplier test statistics provides a principled way for deciding which amongst these two models is more appropriate for the data under analysis [56]. The Lagrange Multiplier test statistics test the significance of the spatial component included in either a spatially lagged model or a spatial error model, with the null hypothesis being that there is no spatial dependency. In our analysis, we consider both non-robust and robust versions of the Lagrange Multiplier statistics [52]. We also compare the *Akaike Information Criterion (AIC)* of each model (ordinary linear regression, spatial lagged model, and spatial error model) to decide the best model in each situation by selecting the model with the lowest AIC value. Comparing the AIC values allows us to easily automate the choice of the best model in each of the several models we build, so in the following sections we will mostly discuss AIC values. However, the results hold also for the Lagrange Multiplier statistics test. In all the spatial models considered in our analysis, we use queen contiguity-based spatial weights.

## Results and discussion

**Fig 2:**
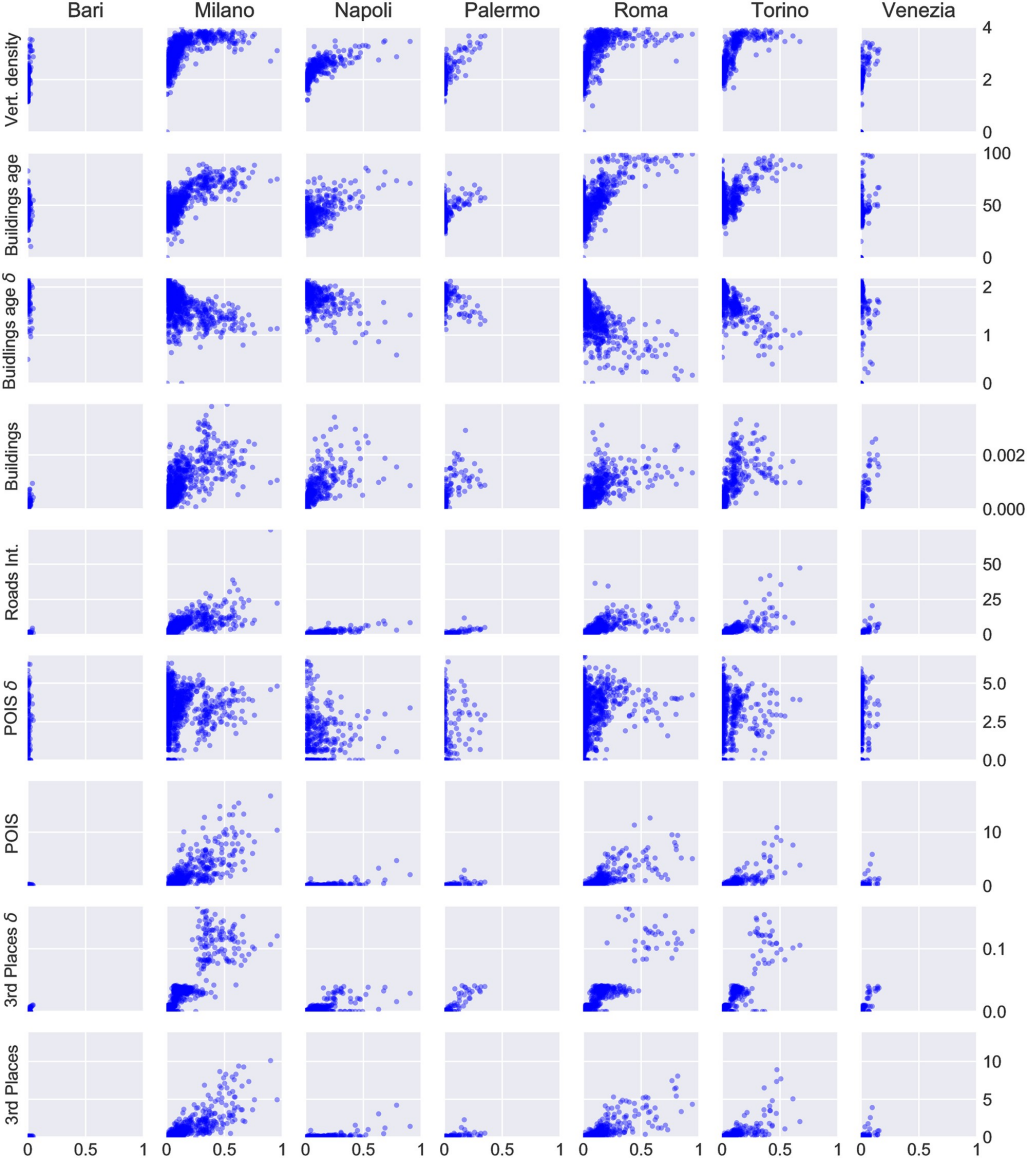
Relationship between presence of people and geographic features. In these scatter plots each point represents a cell in each city. The x-axis encodes the presence of all age groups aggregated together, whereas the y-axis represents the values for each feature described in the Data section. All quantities are normalised by the area of the cell. The points are plotted with some transparency so that accumulation of multiple cells with similar values looks slightly darker.

As an exploratory analysis, we examine the presence of the youngest age group (18 to 30 years old) and the oldest (over 60 years old) in Milan and we observe a similar average pattern over the period of analysis (Fig 1C and 1D). We investigate whether features derived from *OpenStreetMap* and the census are related to the presence of people in different parts of the city. In this first example, visual inspection suggests that the number of road intersections (Fig 1E) and the vertical density (Fig 1F) are higher in areas where there are more people. Fig 2 presents a series of scatter plots showing the relationship between each urban feature and the presence of people.

We then start our investigation by performing a correlation analysis between the mobile phone data and the urban features described in the *Data* and *Data preparation*sections above. For this initial analysis, we consider three different types of aggregation: first, we aggregate all cities together and study the correlation between the presence of different age groups and each urban features; second, we aggregate different age groups and study the correlation across the different cities; and finally, we aggregate all cities and age groups together. As described in the Methods section above, we use *Kendall’s rank correlation coefficient* to measure the association between our variables, and we use *fdr* correction to correct for multiple hypothesis testing [51].

Table 2 reports the results of the correlation analysis, where all values are significant at the 5% level. We find several interesting results. First, if we look at the *aggregated* row, we find that all features are significantly associated with the presence of people. We also note that the diversity of buildings’ ages, i.e. the presence of a mixture of buildings of different ages, is negatively linked with the presence of people. This is in contrast with Jacobs’ theories, and may be due to the unique nature of Italian cities, where city centres primarily consist of old historical buildings. We also note that, perhaps unexpectedly, the diversity of POIs has the smallest association with urban vibrancy. Second, this analysis suggests that the relationship between urban vibrancy and urban features holds consistently across different demographic groups. This provides initial evidence that the urban features which foster vibrancy may be quite universal, in the sense that they create environments which encourage the presence of people of all age groups. Third, we also note that larger cities, in terms of population and number of cells, tend to give rise to larger values of the correlation.

**Table 2:** Investigating the relationship between urban features and vibrancy. We perform an initial correlation analysis, using Kendall’s rank correlation coefficient, between the presence of people and urban features. Here, we present the results when aggregating different cities together, as well as when aggregating different age groups together. All p-values from the correlation tests have been adjusted using *false discovery rate*, and all values are significant (*p* > 0.05).

		Census features				*OpenStreetMap* features				
		Vertical density	Buildings’ ages	Buildings’ ages *δ*	Buildings	Road inters.	POIs *δ*	POIs	3^*rd*^ place *δ*	3^*rd*^ places
Age group	Less than 18	0.59	0.31	-0.41	0.64	0.69	0.12	0.65	0.63	0.64
	18–30	0.55	0.28	-0.38	0.65	0.65	0.09	0.60	0.61	0.61
	31–40	0.56	0.29	-0.38	0.66	0.66	0.08	0.61	0.61	0.61
	41–50	0.58	0.30	-0.39	0.66	0.66	0.09	0.62	0.61	0.62
	51–60	0.59	0.31	-0.41	0.66	0.68	0.10	0.64	0.63	0.63
	Over 60	0.62	0.32	-0.41	0.66	0.70	0.12	0.66	0.65	0.66
City	Milan	0.65	0.50	-0.47	0.60	0.71	0.04	0.71	0.73	0.71
	Rome	0.61	0.42	-0.54	0.65	0.75	0.23	0.75	0.71	0.73
	Palermo	0.39	0.28	-0.23	0.68	0.61	-0.02	0.56	0.41	0.52
	Bari	0.42	0.07	-0.13	0.57	0.59	0.15	0.49	0.47	0.48
	Venice	0.47	0.07	-0.20	0.68	0.72	0.17	0.57	0.56	0.59
	Turin	0.61	0.08	-0.46	0.69	0.74	0.06	0.67	0.65	0.64
	Naples	0.62	0.25	-0.32	0.58	0.68	0.06	0.54	0.40	0.53
	**Aggregated**	0.59	0.31	-0.40	0.65	0.68	0.10	0.64	0.63	0.64

So far, we have analysed the relationship between urban features and vibrancy by either aggregating different age groups together, or different cities. We carry out a further analysis at the city level to investigate whether the same results hold even when considering differences between different urban environments. When analysing data at the city level, we have to carefully consider the data under investigation. Both data on urban features and vibrancy are inherently spatial and, therefore, may exhibit spatial clustering. This means that observations coming from an area are more likely to be similar in value to observations coming from neighbouring areas. This spatial relationship can affect traditional statistical analysis, such as regression models. To demonstrate this with an example, we construct a simple generalised linear model, using a gamma distribution and a logarithmic link function, between the aggregated presence of people and the number of road intersections in the city of Milan. If the data under analysis is spatially correlated, the residuals of such a model would also exhibit such correlation. In order to test for this, we define two cells to be neighbours if they share at least one boundary point. This allows us to test whether neighbouring areas are correlated or not.

We run a *Moran’s I* test on the residuals of the model which confirms the presence of spatial clustering (*Moran’s I* = 0.228; *p* < 0.001; *n* = 921).

In order to address the presence of spatial clustering in the data, we consider spatial regression models [54] as described in the Methods section above. For each age group and city, including the case where we aggregate all the cities together, we calculate the Lagrange Multiplier test statistics to decide whether we should build a regular (i.e. non-spatial) regression model, a spatially lagged model, or a spatial error model. We also calculate the AIC values of the three models. For the majority of the cases the spatial error model performs slightly better than the spatial lag model (0.20% on average) and the regular (non-spatial) regression (0.39% on average). Therefore, to make our presentation easier to follow, here we report results of the spatial error models. However, we report qualitatively similar results of the spatial lag models in section 3 of the S1 File. Each model includes all of the urban features considered above as predictor variables, and the proxy measure of urban vibrancy as dependent variable. Note that, in the case of spatially lagged models, the urban vibrancy measures will also enter the model as predictor variables due to the nature of this model. Before building these models, we standardise each variable by subtracting the mean and dividing by the standard deviation.

**Fig 3:**
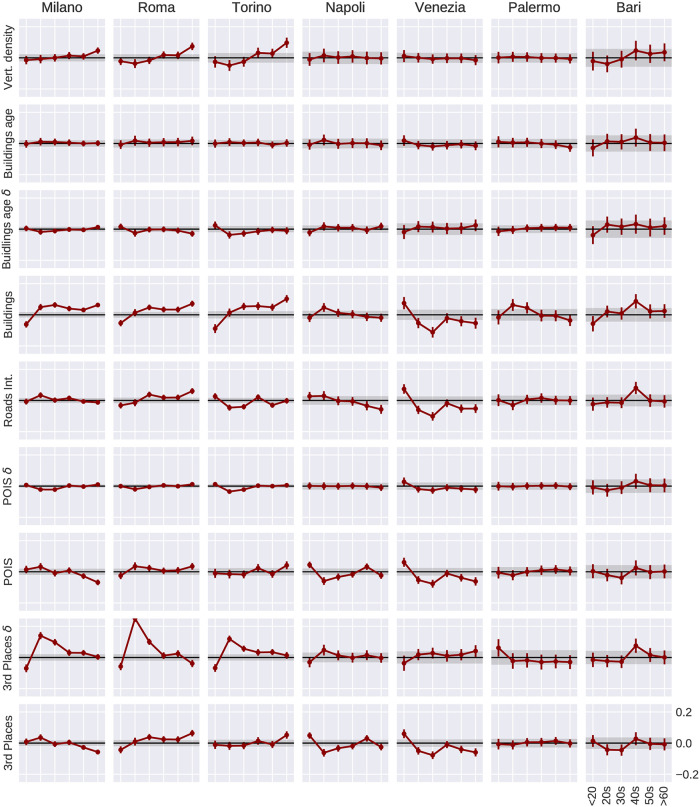
Age group differences in an univariate spatial error model of geographical features. For each city and each age group we run a univariate spatial regression model where the dependent variable Y is the vibrancy, and the independent variable X is one urban feature at a time. We first run the the spatial error model for the aggregate case (all ages) and then subtract the resulting coefficient from each age group. Thus, a positive value for a certain age group indicates that a particular urban feature is relatively more strongly related to the vibrancy compared to the aggregate case. X and Y axes are all the same across small multiples, and labels are only displayed in the bottom-right subplot. Before running the univariate linear model, all values have been standardised subtracting the mean and dividing by the standard deviation. Straight lines along zero indicate no variation across age groups. The error bars are provided by the spatial model result. The grey bands around zero indicate the error of the aggregate value. Milan, Rome and Turin are the cities with higher number of cells (and populations) and more significant patterns. An equivalent plot using the spatial lag model can be found in the S1 File.

Univariate models, with only one urban feature at a time, allow us to test the relationship of each of the geographical features with the vibrancy independently, whereas a multivariate analysis allows to study the set of urban features as a whole. Additionally, they allow us to study the differences across age groups within each city (Fig 3) to investigate whether there are any age dependencies. A first message is that, in smaller cities for which we have fewer cells, no interesting patterns appear, and the model coefficients have relatively large error bars. This suggests that age dependencies may not be detectable at this resolution. As for the most populated and larger cities, such as Milan and Rome, we can see some age-dependent patterns. The first is the positive trend between age groups and the features *vertical density* and *number of buildings*, with the eldest being significantly above the aggregate. The fact that all of them present a similar pattern suggests an influence of the residential structure and may possibly be linked to elderly people living in more central and older buildings in Italian cities. A second pattern is the downward trend (with the exception of the youngest group) for *third place* diversity. Surprisingly, the diversity rather than the density of the *third places* seem to be much more linked to the vibrancy of the younger groups.

Table 3 reports the results of the multivariate analysis at the aggregate level; S1 and S2 Tables in S1 File report the results for the analysis at the city level. Each table contains the coefficients of the model, and coefficients reported in small font size and italic are not significant at the 5% level after correcting using *fdr*. Finally, in each table, we also report the Nagelkerke pseudo R square value, which is a generalisation of the *R*^2^ commonly used in linear regression [57]. For all models, we observe that the Nagelkerke pseudo R square is relatively high, indicating a good relationship between the model variables. We find several results of interest to our analysis. First, broadly speaking we note that the results presented above also hold in this more detailed analysis. Most urban features present a positive association with the presence of people of all age groups. We find again that the diversity of buildings’ ages has a negative relationship. Interestingly, the presence of *third places*, and their diversity, seems to have the strongest effect on vibrancy, as suggested by the largest value of the regression coefficients. This is in agreement with the notion that *third places* are places where people spend their time outside of work and home and which, therefore, are vibrant areas.

**Table 3:** Investigating the spatial relationship between urban features and vibrancy. We model the relationship between the presence of people and urban features by constructing a series of multivariate spatial models to account for spatial correlations present in the data. Here, we present the results of these models when considering all cities together. The table shows the values of the model coefficients, and values are reported in italic and small font size if they are not significant after *fdr* correction (*p* > 0.05). The last column reports the Nagelkerke pseudo R square value, which provides a measure of how good the model is compared to a null model and has a similar interpretation to a traditional regression *R*^2^. Results for the individual cities are presented in S1 and S2 Tables in S1 File.

		Census features				*OpenStreetMap* features					*R*^2^
		Vertical density	Buildings’ ages	Buildings’ ages *δ*	Buildings	Road inters.	POIs *δ*	POIs	3^*rd*^ place *δ*	3^*rd*^ places	
Age group	Less than 18	0.0565	0.0948	-0.0466	-0.0047	0.08642	-0.0550	*-0.0280*	0.3242	0.4155	0.82
	18–30	0.0567	0.0740	*-0.0191*	0.1362	0.0973	-0.0735	-0.0886	0.4386	0.2459	0.79
	31–40	0.0781	0.0768	-0.0368	0.1420	0.0986	-0.0717	-0.1009	0.4029	0.2504	0.80
	41–50	0.1013	0.0796	-0.0491	0.1250	0.1054	-0.0586	-0.0834	0.3603	0.2732	0.80
	51–60	0.1085	0.0890	-0.0644	0.0999	0.0845	-0.0591	-0.0748	0.3888	0.2869	0.83
	Over 60	0.1865	0.0728	-0.0713	0.1449	0.0763	-0.0589	-0.0727	0.3600	0.2513	0.87
City	Milan	*0.0053*	0.0176	-0.0103	*-0.0005*	0.0199	-0.0121	*-0.0010*	0.0732	0.0818	0.89
	Rome	0.0171	0.0187	*-0.0058*	*-0.0005*	*-0.0037*	-0.0076	*-0.0042*	0.0781	0.0539	0.90
	Turin	0.0247	0.0092	*-0.0062*	0.0132	0.0252	-0.0123	0.0689	0.0304	-0.0403	0.90
	Naples	0.0264	0.0205	*-0.0094*	0.0126	0.0627	-0.0274	0.0899	0.0759	-0.0502	0.92
	Venice	0.0022	*-0.0007*	*-0.0007*	0.0072	0.0067	-0.0023	*-0.0046*	*0.0096*	0.0045	0.92
	Palermo	0.0026	0.0064	-0.0047	0.0086	0.0389	-0.0055	*-0.0129*	0.0511	*-0.0146*	0.92
	Bari	0.0026	*-0.0001*	-0.0018	0.0027	*0.0005*	-0.0016	*-0.0006*	0.0036	*0.0014*	0.83
	**Aggregated**	0.0893	0.0878	-0.0486	0.0761	0.0927	-0.0613	*-0.0649*	0.3708	0.3351	0.84

This finding provides further evidence that the notion of *third places* is indeed crucial when trying to understand what makes urban environments vibrant. So far, we have aggregated together all *third places*, but we now only consider each of the four categories described in the Data section above. Each of these four categories enters our models as an independent variable. We use the same modelling procedure as above, by calculating the Lagrange Multiplier test statistics and the AIC values to select the appropriate model for each case. In this case spatial lag model performs better than the spatial error model (1.64% on average) and much better than regular regression (9.37% on average). For coherence with the previous results and ease of interpretability, Table 4 shows the results of the spatial error model while the spatial lag can be found in section 3 of the S1 File. As in our previous analysis, coefficients which are not significant are reported in italic and smaller font size. First, we observe that the pseudo *R*^2^ of these models is broadly comparable in magnitude with that of the models presented above. This reasserts the idea that *third places* play a fundamental role in making urban environments vibrant. We also note that the category *commercial venue* tends to have the largest coefficients, indicating a stronger importance in the relationship with urban vibrancy. Finally, we again find that the relationship broadly holds across all age groups in a similar fashion, thus showing no strong differences between different age groups.

**Table 4:** Investigating the role of *third places* on urban vibrancy. Further to the analysis above, we investigate in more detail the relationship between each *third places* category and urban vibrancy. As before, we construct a range of multivariate spatial models and present the results in this table. The table shows the values of the model coefficients, and values are reported in italic and small font size if they are not significant after *fdr* correction (*p* > 0.05). The last column reports the Nagelkerke pseudo R square value.

		Third places				R^2^
		Organised activity	Eating, drinking, talking	Outdoor	Commercial venue	
Age group	Less than 18	0.1814	0.1158	0.0927	0.3607	0.75
	18–30	0.1357	0.1398	0.1171	0.2149	0.66
	31–40	0.1357	0.1147	0.1347	0.2230	0.68
	41–50	0.1520	0.1142	0.1395	0.2466	0.70
	51–60	0.1642	0.1478	0.1440	0.2273	0.72
	Over 60	0.1112	0.1877	0.1265	0.1953	0.74
City	Milan	0.0270	0.0454	*0.0030*	0.0509	0.80
	Rome	0.0341	*-0.0021*	0.0072	0.0668	0.82
	Turin	0.0199	0.0123	*0.0067*	0.0358	0.73
	Naples	0.0539	*0.0031*	0.0491	0.0464	0.70
	Venice	0.0114	-0.0102	0.0050	0.0091	0.83
	Palermo	0.0187	0.0336	*0.0035*	*-0.0214*	0.72
	Bari	0.0032	*-0.0006*	*-0.0007*	0.0024	0.70
	**Aggregated**	0.1598	0.1334	0.1189	0.2855	0.75

**Fig 4:**
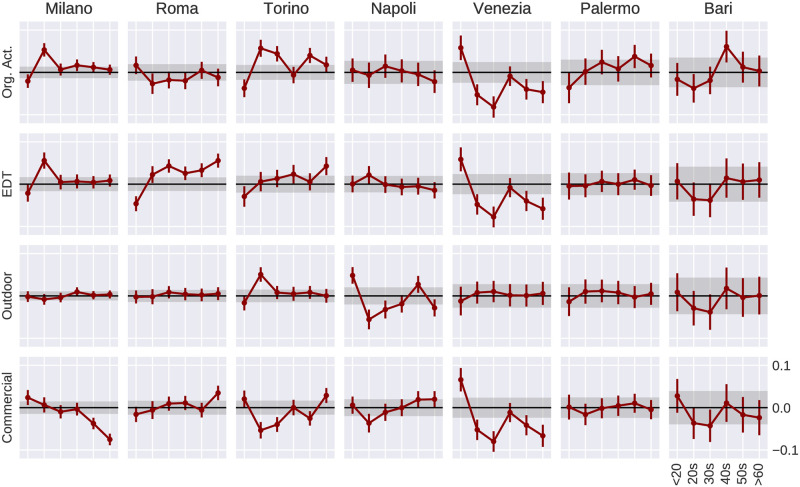
Age group differences in an univariate spatial error model of *third places*. For each city and each age group we run a univariate spatial error model where the dependent variable Y is the vibrancy, and the independent variable X is one of the categories of *third places* at a time. Similar to Fig 3, we firstly run the same model for the aggregated case (all ages) and subtract the resulting coefficient from each age group. X and Y axes are all the same across small multiples, and labels are only displayed in the bottom-right subplot. All values have been standardised subtracting the mean and dividing by the standard deviation before running the univariate model. Straight lines along zero indicate no variation across age groups. The error bars are provided by the spatial model result. The grey bands around zero indicate the error of the aggregate value. Milan, Rome and Turin are the cities with higher number of cells (and populations) and more significant patterns. An equivalent plot using the spatial lag model can be found in the S1 File.

Finally, we also study those four components independently by means of running a univariate analysis and computing the difference of each age group with respect to the aggregate. Fig 4 reports such differences by age. Although we can see a few significant differences on some of the categories and cities (for example a spike for the 20–30 y.o. in Milan’s“Eating-Drinking-Talking” category) we do not observe any general pattern of notice. This stresses once more the importance of the general variety over the total count of one sub-type of *third places*.

To further test the robustness of our results, we perform a final validation step. We repeat the same correlation analysis presented earlier, but we randomise the presence data by reshuffling it. This randomisation should remove any existing pattern in the data and, therefore, no correlation should be found. If a significant correlation were found, this would suggest that our results may be due to chance alone, since a randomised version of the data also exhibits a correlation. For each city and age group, we calculate the correlation between each urban feature and 1,000 randomised versions of the presence data. We then adjust all resulting p-values using *fdr* correction as above, and count how many correlations are found to be significant. We find that no correlation is significant after the randomisation of the data. This provides further evidence that our results are not due to chance alone and that the relationship which we observed indicates a genuine effect present in the data.

We also want to further investigate whether the effects that we observe in the analysis presented above could be simply due to the population which lives in the different parts of each city, rather than the spontaneous interactions and presence of people making the city vibrant. In order to do so, we extract data from the census on the number of people of different age groups living in each of the cell under analysis, and we then reproduce the exact same analysis presented above. Even though the age groups provided by the Italian census do not perfectly match that of the presence data, we consider the categories to be similar enough to allow this to be a valid comparison. S7-S10 Tables in S1 File present the results of this analysis. Whilst broadly speaking there still is a relationship between urban features and population, we note that the strongest effect is not given by *third places* anymore, but from features related to buildings instead. This provides further evidence that using data from mobile phone allows us to better understand what makes an urban environment vibrant not only in terms of the people living there, but instead also due to the people who spend time in different parts of a city. In fact, our analysis really highlights the role that *third places* play in making an urban environment vibrant.

## Conclusion

Our study focused on the relationship between urban vibrancy and features of the urban environment across seven large metropolitan cities in Italy. Our contribution explicitly considers spatial effects in the data, which so far have been overlooked in part of the computational social science literature. We have considered features from the urban vibrancy literature and have investigated whether their relationship with the presence of people holds across different age groups. To measure urban vibrancy, we have used a large data set containing the presence of mobile phone users of different age groups, and we have extracted a range of urban features from *OpenStreetMap* and the Italian 2011 census. We have found several results of interest. First, we have shown how geographical features extracted from a crowdsourcing platform are related to urban vibrancy even when taking spatial effects into account. Although future work would need to explore these results in cities in other countries and continents, our analyses are able to model vibrancy in Italian cities with high values of pseudo-*R*^2^. Second, we have identified the importance of *third places* in understanding vibrant environments. By considering only the four categories of *third places*, we have been able to model urban vibrancy with a pseudo-*R*^2^ comparable to that of models including several other geographical features. Finally, we observe a decrease with age in the strength of the relationship between vibrancy by age group and *third place* diversity, with the 18–30 year old age group exhibiting higher vibrancy in areas with higher diversity of *third places*. However, despite our initial hypothesis that age may be a relevant factor when studying vibrancy, we have not found any other general evidence to support this across several urban features.

Our analysis has also explicitly taken into account the spatial effects present in the data. This issue has often been overlooked in parts of the computational social science literature, and we believe that the approach presented here should become more prominent in this research area.

It is also important to acknowledge that there are several limitations to our study. First, we rely on mobile phone data as a proxy for urban vibrancy. More precisely, we measure vibrancy using the presence of mobile phone users as derived by *Telecom Italia* from the number of outgoing calls in their cellular network. This clearly does not capture the full population of mobile phone users, and it is not guaranteed to be an unbiased sample of the general population. Having access to mobile phone data for a longer time period, or for more providers, would greatly improve the analysis. Additionally, whilst mobile phone data have been used in several studies as a proxy for urban vibrancy, it is important to acknowledge that people living in each area also contribute to generating mobile phone data even though they may not necessarily contribute to making the environment vibrant. Future studies should focus on how we can disentangle these two effects present in the data. Secondly, some of the urban features in our analyses are derived from data available on *OpenStreetMap*. This is a volunteer-based, crowdsourced mapping tool, which is not necessarily complete nor accurate. Furthermore, the *OpenStreetMap* data was collected in a different time period from the mobile phone data, and similarly for the census data. Whilst urban features and census data may not change over short periods of time, this is undoubtedly a limitation of our analysis. The mobile phone data used in our analysis was also spatially aggregated by *Telecom Italia* to spatial cells. Due to this, we aggregated urban features to the same level. However, in dense metropolitan cities, this aggregation may result in a cell which contains a very diverse range of features, including many related to different social activities. A more spatially granular analysis would allow us to better investigate which activities and features promote vibrancy. Finally, our study has not considered any differences in urban versus suburban areas, or any socioeconomic factors. Whilst this was a deliberate choice to focus only on features of the built urban environment, socioeconomic factors are likely to contribute to urban vibrancy.

Our study has considered urban vibrancy as an aggregated measure over the period of analysis. However, future studies may wish to extend this to further investigate temporal differences. For example, different areas may be more or less vibrant at different times of the day or week, and this may be linked to demographic characteristics of the people living in those environment. Finally, the nature and direction of any link observed in our analysis requires further careful investigation.

Future studies may want to consider a broader range of geographical features. For example, accessibility to parks or public transport might be related to how vibrant an environment is. Additionally, future research should also incorporate socioeconomic factors in the analysis to investigate whether they have a significant effect in making different urban environments vibrant. Further work also needs to be done to investigate whether more accurate or complete proxies for urban vibrancy can be derived from existing or new data sets, so that vibrancy can be investigated in all its diverse aspects.

Finally, our analysis shows how data derived from our interactions with the mobile phone network, as well as crowdsourced data on cities, can be used to better understand the environment in which we live in. These findings may be useful in better understanding and designing cities.

## Supporting information

S1 File (PDF)

## References

[1] DESA U. World Urbanization Prospects: 2018. New York: United Nations. 2014;.

[2] Bettencourt LM, Lobo J, Helbing D, Kühnert C, West GB. Growth, innovation, scaling, and the pace of life in cities. Proceedings of the national academy of sciences. 2007;104(17):7301–7306. 10.1073/pnas.0610172104 17438298 PMC1852329

[3] Glaeser E. Triumph of the City: How Our Greatest Invention Makes Us Richer, Smarter, Greener, Healthier, and Happier, 2011; 2011.

[4] Musterd S, Ostendorf W. Urban segregation and the welfare state: Inequality and exclusion in western cities. Routledge; 2013.

[5] Glaeser EL, Resseger M, Tobio K. Inequality in cities. Journal of Regional Science. 2009;49(4):617–646. 10.1111/j.1467-9787.2009.00627.x

[6] Zheng Y, Capra L, Wolfson O, Yang H. Urban computing: concepts, methodologies, and applications. ACM Transactions on Intelligent Systems and Technology (TIST). 2014;5(3):1–55.

[7] Blondel VD, Decuyper A, Krings G. A survey of results on mobile phone datasets analysis. EPJ Data Science. 2015;4:10. 10.1140/epjds/s13688-015-0046-0

[8] Barbosa H, Barthelemy M, Ghoshal G, James CR, Lenormand M, Louail T, et al. Human mobility: Models and applications. Physics Reports. 2018;734:1–74. 10.1016/j.physrep.2018.01.001

[9] OpenStreetMap contributors. Planet dump retrieved from https://planet.osm.org; 2017. https://www.openstreetmap.org.

[10] Lazer D, Pentland AS, Adamic L, Aral S, Barabasi AL, Brewer D, et al. Computational social science. Science. 2009;323:721–723. 10.1126/science.1167742 19197046 PMC2745217

[11] Vespignani A. Predicting the behavior of techno-social systems. Science. 2009;325:425–428. 10.1126/science.1171990 19628859

[12] Botta F, Preis T, Moat HS. In search of art: rapid estimates of gallery and museum visits using Google Trends. EPJ Data Science. 2020;9(1):14. 10.1140/epjds/s13688-020-00232-z

[13] King G. Ensuring the data-rich future of the social sciences. Science. 2011;331:719–721. 10.1126/science.1197872 21311013

[14] Botta F, Moat HS, Preis T. Quantifying crowd size with mobile phone and Twitter data. Royal Society Open Science. 2015;2:150162. 10.1098/rsos.150162 26064667 PMC4453255

[15] Botta F, Moat HS, Preis T. Measuring the size of a crowd using Instagram. Environment and Planning B: Urban Analytics and City Science. 2019; p. 2399808319841615.

[16] Quercia D, O’Hare NK, Cramer H. Aesthetic capital: what makes London look beautiful, quiet, and happy? In: Proceedings of the 17th ACM conference on Computer supported cooperative work & social computing; 2014. p. 945–955.

[17] Salesses P, Schechtner K, Hidalgo CA. The collaborative image of the city: mapping the inequality of urban perception. PloS one. 2013;8(7):e68400. 10.1371/journal.pone.0068400 23894301 PMC3722224

[18] Aiello LM, Schifanella R, Quercia D, Aletta F. Chatty maps: constructing sound maps of urban areas from social media data. Royal Society Open Science. 2016;3:150690. 10.1098/rsos.150690 27069661 PMC4821272

[19] Quercia D, Aiello LM, Mclean K, Schifanella R. Smelly maps: the digital ife of urban smellscapes. In: AAAI Publications; 2015. p. 327–336.

[20] Quercia D, Schifanella R, Aiello LM. The shortest path to happiness. In: Proceedings of the 25th ACM conference on Hypertext and social media—HT’14; 2014. p. 116–125.

[21] Seresinhe CI, Preis T, Moat HS. Quantifying the link between art and property prices in urban neighbourhoods. Royal Society Open Science. 2016;3:160146. 10.1098/rsos.160146 27152228 PMC4852651

[22] Seresinhe CI, Preis T, Moat HS. Quantifying the impact of scenic environments on health. Scientific Reports. 2015;5:16899. 10.1038/srep16899 26603464 PMC4658473

[23] Porzi L, Rota Bulò S, Lepri B, Ricci E. Predicting and understanding urban perception with convolutional neural networks. In: Proceedings of the 23rd ACM international conference on Multimedia; 2015. p. 139–148.

[24] Law S, Seresinhe CI, Shen Y, Gutierrez-Roig M. Street-Frontage-Net: urban image classification using deep convolutional neural networks. International Journal of Geographical Information Science. 2018; p. 1–27.

[25] Seresinhe CI, Preis T, Moat HS. Using deep learning to quantify the beauty of outdoor places. Royal Society open science. 2017;4(7):170170. 10.1098/rsos.170170 28791142 PMC5541537

[26] Jacobs J. The death and life of American cities. Random House; 1961.

[27] Gehl J. Life between buildings: using public space. Island Press; 1971.

[28] De Nadai M, Staiano J, Larcher R, Sebe N, Quercia D, Lepri B. The death and life of great Italian cities: a mobile phone data perspective. In: Proceedings of the 25th international conference on world wide web. International World Wide Web Conferences Steering Committee; 2016. p. 413–423.

[29] Sung H, Lee S, Cheon S. Operationalizing jane jacobs’s urban design theory: Empirical verification from the great city of seoul, korea. Journal of Planning Education and Research. 2015;35(2):117–130. 10.1177/0739456X14568021

[30] Blessing R. Jane eternal: The lasting influence of Jane Jacobs’s death and life of great American cities on urban planning; 2017.

[31] King K. Jane Jacobs and ‘the need for aged buildings’: Neighbourhood historical development pace and community social relations. Urban Studies. 2013;50(12):2407–2424. 10.1177/0042098013477698 PMC3808089 24163485

[32] Grant JL. Time, Scale, and Control: How New Urbanism Mis (Uses) Jane Jacobs. In: Reconsidering Jane Jacobs. Routledge; 2017. p. 91–176.

[33] Oldenburg R, Brissett D. The third place. Qualitative sociology. 1982;5(4):265–284. 10.1007/BF00986754

[34] Oldenburg R. The great good place: Cafés, coffee shops, community centers, beauty parlors, general stores, bars, hangouts, and how they get you through the day. Paragon House Publishers; 1989.

[35] Huang B, Zhou Y, Li Z, Song Y, Cai J, Tu W. Evaluating and characterizing urban vibrancy using spatial big data: Shanghai as a case study. Environment and Planning B: Urban Analytics and City Science. 2019; p. 2399808319828730.

[36] Kim YL. Seoul’s Wi-Fi hotspots: Wi-Fi access points as an indicator of urban vitality. Computers, Environment and Urban Systems. 2018;72:13–24. 10.1016/j.compenvurbsys.2018.06.004

[37] Sulis P, Manley E, Zhong C, Batty M. Using mobility data as proxy for measuring urban vitality. Journal of Spatial Information Science. 2018;16:137–162.

[38] Wu C, Ye X, Ren F, Du Q. Check-in behaviour and spatio-temporal vibrancy: An exploratory analysis in Shenzhen, China. Cities. 2018;77:104–116. 10.1016/j.cities.2018.01.017

[39] Yue Y, Zhuang Y, Yeh AG, Xie JY, Ma CL, Li QQ. Measurements of POI-based mixed use and their relationships with neighbourhood vibrancy. International Journal of Geographical Information Science. 2017;31(4):658–675. 10.1080/13658816.2016.1220561

[40] Long Y, Huang C. Does block size matter? The impact of urban design on economic vitality for Chinese cities. Environment and Planning B: Urban Analytics and City Science. 2019;46(3):406–422.

[41] Ye Y, Li D, Liu X. How block density and typology affect urban vitality: An exploratory analysis in Shenzhen, China. Urban Geography. 2018;39(4):631–652. 10.1080/02723638.2017.1381536

[42] Humphrey C, Jensen ST, Small DS, Thurston R. Urban vibrancy and safety in Philadelphia. Environment and Planning B: Urban Analytics and City Science. 2017; p. 2399808319830403.

[43] Delclòs-Alió X, Gutiérrez A, Miralles-Guasch C. The urban vitality conditions of Jane Jacobs in Barcelona: Residential and smartphone-based tracking measurements of the built environment in a Mediterranean metropolis. Cities. 2019;86:220–228. 10.1016/j.cities.2018.09.021

[44] Bogomolov A, Lepri B, Staiano J, Letouzé E, Oliver N, Pianesi F, et al. Moves on the street: Classifying crime hotspots using aggregated anonymized data on people dynamics. Big data. 2015;3(3):148–158. 10.1089/big.2014.0054 27442957

[45] Traunmueller M, Quattrone G, Capra L. Mining mobile phone data to investigate urban crime theories at scale. In: International Conference on Social Informatics. Springer; 2014. p. 396–411.

[46] Source of the Dataset: TIM Big Data Challenge 2015, www.telecomitalia.com/bigdatachallenge;.

[47] https://www.istat.it/it/archivio/222527.

[48] http://download.geofabrik.de.

[49] Shannon CE. A mathematical theory of communication. Bell system technical journal. 1948;27(3):379–423. 10.1002/j.1538-7305.1948.tb01338.x

[50] Jeffres LW, Bracken CC, Jian G, Casey MF. The impact of third places on community quality of life. Applied Research in Quality of Life. 2009;4(4):333. 10.1007/s11482-009-9084-8

[51] Benjamini Y, Hochberg Y. Controlling the false discovery rate: a practical and powerful approach to multiple testing. Journal of the Royal Statistical Society Series B. 1995;57:289–300.

[52] Anselin L, Syabri I, Kho Y. GeoDa: an introduction to spatial data analysis. In: Handbook of applied spatial analysis. Springer; 2010. p. 73–89.

[53] Anselin L. Spatial econometrics: methods and models. vol. 4. Springer Science & Business Media; 2013.

[54] Ward MD, Gleditsch KS. Spatial regression models. vol. 155. Sage Publications; 2018.

[55] Anselin L. Under the hood issues in the specification and interpretation of spatial regression models. Agricultural economics. 2002;27(3):247–267. 10.1111/j.1574-0862.2002.tb00120.x

[56] Anselin L. Lagrange multiplier test diagnostics for spatial dependence and spatial heterogeneity. Geographical analysis. 1988;20(1):1–17. 10.1111/j.1538-4632.1988.tb00159.x

[57] Nagelkerke NJ, et al. A note on a general definition of the coefficient of determination. Biometrika. 1991;78(3):691–692. 10.1093/biomet/78.3.691

