# Supplementary material for: Modelling urban vibrancy with mobile phone and OpenStreetMap data
Source: PLoS One. 2021 Jun 2;16(6):e0252015. doi: 10.1371/journal.pone.0252015 (PMC8172046; doi:10.1371/journal.pone.0252015)
Supplement: S1 File — (PDF) [file pone.0252015.s001.pdf]

Modelling urban vibrancy with mobile phone and *OpenStreetMap* data

Supplementary Material

Federico Botta[1*†], Mario Gutiérrez-Roig[2†]

1 Department of Computer Science, University of Exeter, Exeter, UK
2 Department of Mathematical Science, University of Essex, Colchester, UK

* f.botta@exeter.ac.uk
† These authors contributed equally.

1 Cities under analysis

In Figs S1 and S2 we depict the areas under analysis for the cities not shown in Fig 1 of the *Main Text*.

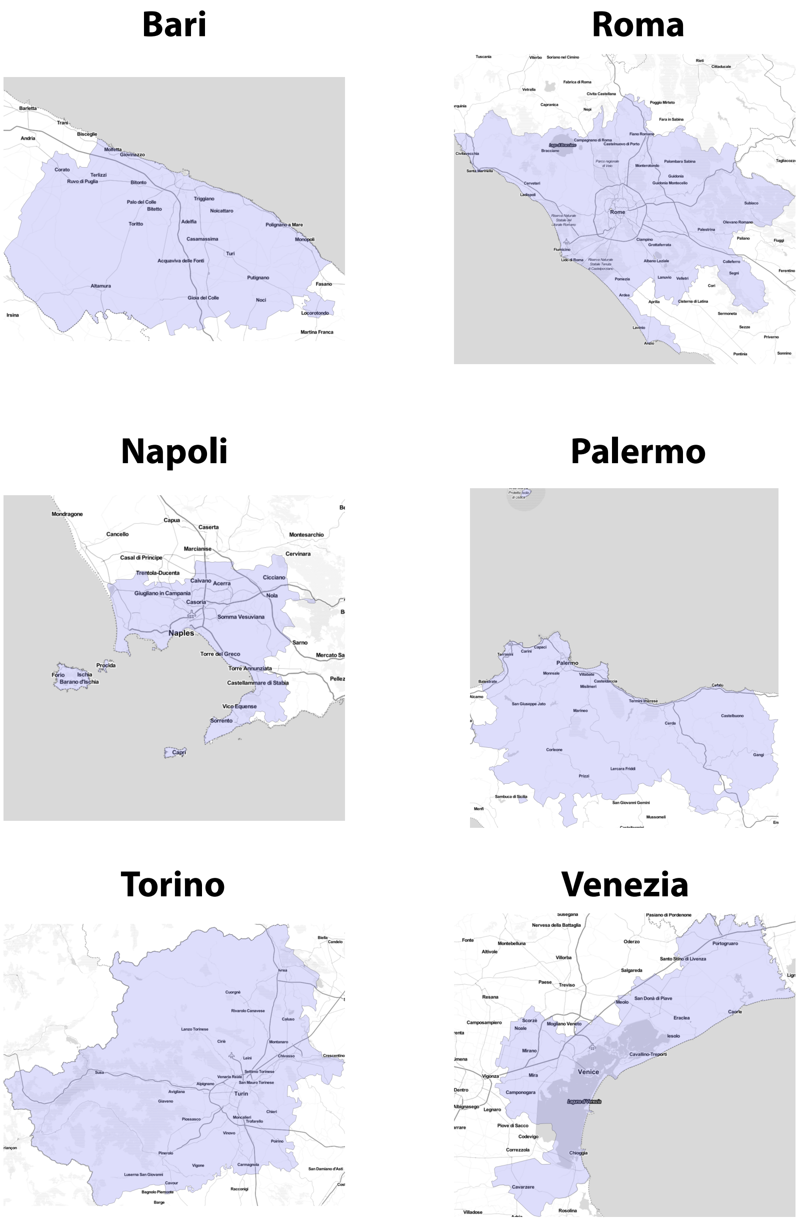

Figure S1. Metropolitan cities under analysis | We present here the metropolitan cities which are included in our analysis, with the exception of Milan which was already presented in the *Main Text*.

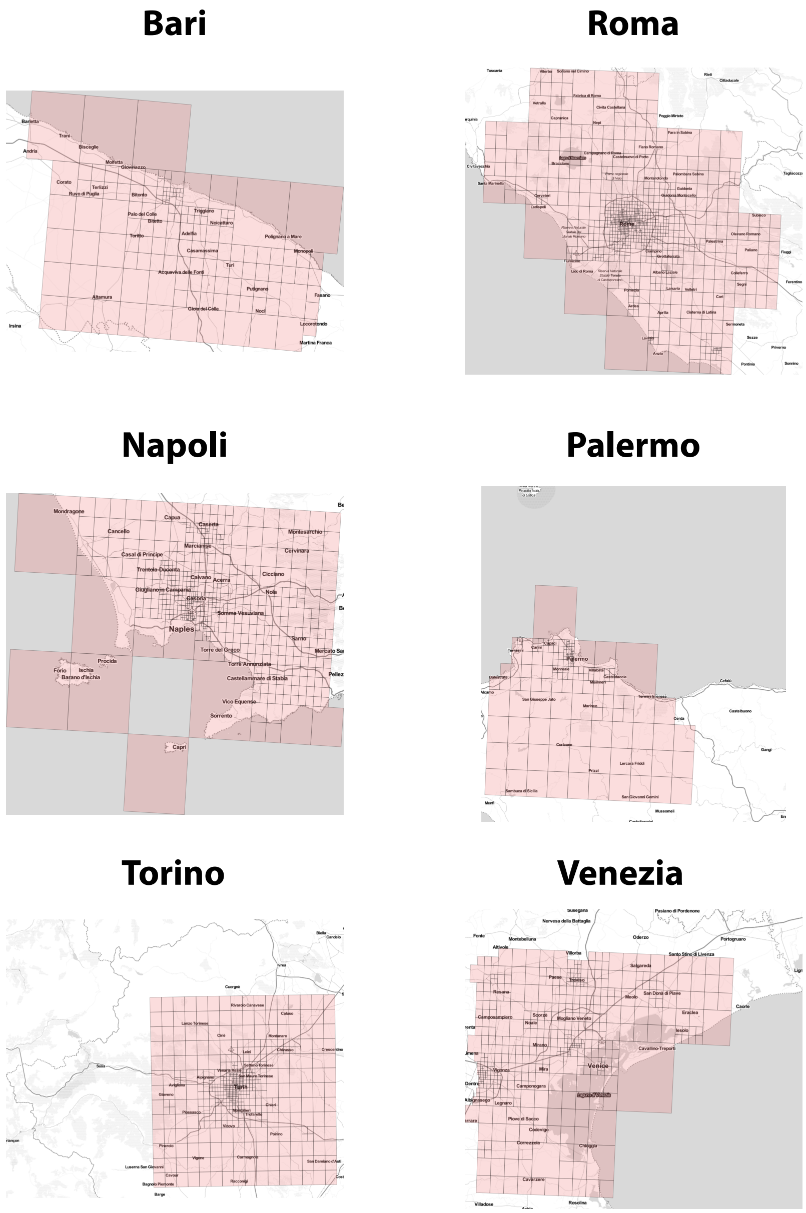

Figure S2. Grids of mobile phone network for the metropolitan cities under analysis | We present here the spatial grids for which we retrieve mobile phone data in our analysis for the metropolitan cities which we investigate, with the exception of Milan which was already presented in the *Main Text*.

2 Multivariate analysis by city

We present here the results of the multivariate spatial analysis at the individual city level. The aggregate results have been presented and discusses in the *Main Text*.

Table S1. See the caption of Table 3 in the *Main Text*.

		Census features				*OpenStreetMap*					
	Age	Vertical density	Buildings age	Buildings age δ	Buildings	Roads inters.	POIs δ	POIs	$3^{rd} places \delta$	$3^{rd} places$	R^2
Milano	Less than 18	*-0.00104*	0.00921	-0.00457	-0.00781	0.00824	-0.00483	*0.00543*	0.02702	0.04278	0.83
	18-30	*0.00043*	0.00121	-0.00065	0.00111	0.00183	-0.00155	-0.00228	0.00874	0.00818	0.83
	31-40	0.00083	0.00121	-0.00092	0.00137	0.00184	-0.00162	*-0.00126*	0.00841	0.00697	0.86
	41-50	0.00111	0.00177	-0.00141	0.00119	0.00295	-0.00153	*-0.00052*	0.00978	0.00896	0.87
	51-60	0.00122	0.00180	-0.00133	0.00087	0.00243	-0.00141	*-0.00002*	0.00922	0.00709	0.89
	Over 60	0.00326	0.00233	-0.00145	0.00264	0.00197	-0.00107	*-0.00169*	0.00909	0.00639	0.90
Roma	Less than 18	0.00384	0.00927	*-0.00202*	-0.00503	*-0.00248*	-0.00279	*-0.00782*	0.03914	0.03359	0.78
	18-30	0.00084	0.00078	*-0.00041*	*-0.00018*	*-0.00040*	-0.00059	0.00268	0.00659	*0.00044*	0.75
	31-40	0.00119	0.00116	*-0.00030*	0.00041	*0.00027*	-0.00080	*0.00016*	0.00708	0.00363	0.81
	41-50	0.00275	0.00215	-0.00079	0.00091	*-0.00009*	-0.00105	*-0.00066*	0.00957	0.00661	0.81
	51-60	0.00277	0.00235	-0.00090	0.00080	*-0.00011*	-0.00110	*-0.00081*	0.00974	0.00666	0.81
	Over 60	0.00472	0.00183	*-0.00143*	*0.00286*	*0.00016*	-0.00113	*0.00089*	0.00619	0.00442	0.83
Palermo	Less than 18	*-0.00008*	0.00230	*-0.00153*	*0.00159*	0.01384	*-0.00143*	*-0.00514*	0.01623	*-0.00490*	0.70
	18-30	*0.00037*	0.00090	-0.00076	0.00179	0.00462	-0.00074	*-0.00107*	0.00593	*-0.00182*	0.75
	31-40	*0.00025*	0.00078	*-0.00047*	0.00155	0.00437	-0.00066	*-0.00140*	0.00544	*-0.00153*	0.76
	41-50	*0.00027*	0.00116	*-0.00071*	0.00130	0.00726	*-0.00082*	*-0.00228*	0.00808	*-0.00290*	0.73
	51-60	*0.00044*	0.00098	*-0.00060*	0.00114	0.00553	-0.00078	*-0.00141*	0.00735	*-0.00244*	0.73
	Over 60	*0.00054*	*0.00081*	*-0.00053*	*0.00084*	0.00576	-0.00085	*-0.00131*	0.00758	-0.00300	0.71

Table S2. See the caption of Table 3 in the *Main Text*.

	Age	Census features				*OpenStreetMap*					
		Vertical density	Buildings age	Buildings age δ	Buildings	Roads inters.	POIs δ	POIs	$3^{rd} places \delta$	$3^{rd} places$	R^2
Bari	Less than 18	0.00107	*0.00000*	-0.00081	0.00073	0.00028	*-0.00062*	*-0.00023*	0.00153	*0.00065*	0.65
	18-30	0.00029	*-0.00001*	-0.00017	0.00031	*0.00010*	-0.00021	*0.00012*	0.00041	*-0.00007*	0.74
	31-40	0.00033	*-0.00001*	-0.00020	0.00038	*0.00007*	-0.00020	*-0.00006*	0.00045	*0.00006*	0.73
	41-50	0.00038	*0.00002*	-0.00022	0.00043	0.00022	-0.00022	*-0.00003*	0.00059	*0.00002*	0.70
	51-60	0.00034	*0.00001*	-0.00022	0.00039	*-0.00001*	-0.00020	*-0.00009*	0.00049	*0.00018*	0.69
	Over 60	0.00030	*0.00001*	-0.00019	0.00035	*0.00000*	-0.00019	*-0.00006*	0.00040	*0.00016*	0.70
Venezia	Less than 18	*0.00052*	*0.00025*	*-0.00047*	0.00358	0.00436	-0.00070	*-0.00538*	0.00379	*0.00427*	0.80
	18-30	0.00038	*-0.00017*	*-0.00009*	0.00110	0.00101	-0.0004	*-0.00139*	0.00132	*0.00081*	0.82
	31-40	0.00037	-0.00019	*-0.00009*	0.00088	0.00084	-0.00039	*-0.00055*	0.00121	*0.00004*	0.80
	41-50	0.00043	*-0.00018*	*-0.00012*	0.00124	0.00123	-0.00044	*-0.00144*	0.00155	*0.00087*	0.84
	51-60	0.00038	*-0.00012*	*-0.00013*	0.00124	0.00118	-0.00044	*-0.00100*	0.00153	*0.00034*	0.83
	Over 60	0.00037	*-0.00013*	*-0.00011*	0.00130	0.00168	-0.00048	-0.00217	0.00202	*0.00111*	0.81
Torino	Less than 18	0.00925	0.00413	*-0.00268*	*0.00026*	0.01325	-0.00431	0.03019	0.01028	-0.01760	0.83
	18-30	0.00191	0.00072	-0.0007	0.00158	0.00116	-0.00152	0.00713	0.00429	-0.00375	0.81
	31-40	0.00214	0.00074	-0.00077	0.00212	0.00197	-0.00151	0.00659	0.0039	-0.00413	0.85
	41-50	0.00321	0.00087	*-0.00061*	0.00297	0.00311	-0.00158	0.00849	0.00415	-0.00527	0.86
	51-60	0.00314	0.00108	*-0.00064*	0.0025	0.00282	-0.00150	0.00785	0.00409	-0.00456	0.86
	Over 60	0.00458	0.00111	*-0.00027*	0.00439	0.00230	-0.00167	0.00915	0.00332	-0.00521	0.87
Napoli	Less than 18	0.00746	0.00590	-0.00354	0.00276	0.01986	-0.00811	0.02644	0.01719	*-0.00948*	0.68
	18-30	0.00292	0.00258	*-0.00101*	0.00262	0.00902	-0.00347	0.00954	0.01246	-0.00743	0.67
	31-40	0.00353	0.00281	-0.00129	0.00221	0.00933	-0.00386	0.01072	0.01184	-0.00693	0.68
	41-50	0.00490	0.00353	*-0.00153*	0.00268	0.01126	-0.00466	0.01702	0.01452	-0.01131	0.68
	51-60	0.00373	0.00305	-0.00140	0.00140	0.00751	-0.00383	0.01411	0.01070	-0.00726	0.67
	Over 60	0.00322	0.00285	*-0.00103*	*0.00073*	0.00592	-0.00296	0.01119	0.00948	-0.00716	0.67

3 Analysis using census data

Tables S3, S4, S5 and S6 show the results of the same analysis presented in the *Main Text* when using population data derived from the census as our dependent variable instead of the mobile phone data.

Table S3. Investigating the relationship between urban features and population. This table contains the correlation, calculated using Kendall's correlation coefficient, between urban features and different age groups of the population as measured by the Italian census. All values are significant at the 5% level, after adjusting for multiple comparisons using *false discovery rate* correction.

		Census features				*OpenStreetMap*				
		Vertical density	Buildings age	Buildings age δ	Buildings	Roads inters.	POIs δ	POIs	$3^{rd} places \delta$	$3^{rd} places$
Age group	Less than 20	0.50	0.18	0.05	0.59	0.55	0.39	0.55	0.44	0.60
	20-29	0.50	0.19	0.05	0.60	0.55	0.39	0.55	0.43	0.60
	30-39	0.50	0.18	0.04	0.60	0.56	0.39	0.56	0.44	0.60
	40-49	0.48	0.18	0.04	0.60	0.56	0.38	0.55	0.43	0.60
	50-59	0.48	0.19	0.04	0.61	0.56	0.38	0.55	0.44	0.60
	Over 60	0.46	0.21	0.06	0.63	0.56	0.40	0.56	0.43	0.60
City	Milano	0.59	0.41	-0.31	0.67	0.61	0.13	0.63	0.60	0.66
	Roma	0.62	0.28	-0.39	0.70	0.67	0.30	0.65	0.62	0.66
	Palermo	0.47	0.33	-0.11	0.73	0.70	0.12	0.63	0.47	0.61
	Bari	0.49	0.19	0.05	0.61	0.56	0.39	0.55	0.44	0.60
	Venezia	0.54	0.12	-0.26	0.66	0.65	0.26	0.63	0.50	0.63
	Torino	0.60	0.03	-0.33	0.79	0.70	0.18	0.69	0.64	0.67
	Napoli	0.55	0.27	-0.24	0.65	0.61	0.21	0.54	0.41	0.55
	**Aggregated**	0.59	0.26	-0.30	0.72	0.65	0.21	0.62	0.59	0.64

Table S4. Investigating the spatial relationship between urban features and population. We model the relationship between the population, as measured by the census, and urban features by constructing a series of multivariate spatial models to account for spatial correlations present in the data. Here, we present the results of these models when considering all cities together. The table shows the values of the model coefficients, and values are reported in italic if they are not significant after *fdr* correction ($p > 0.05$). The last column reports the Nagelkerke pseudo R square value, which provides a measure of how good the model is compared to a null model and has a similar interpretation to a traditional regression R^2. Results for the individual cities are presented in Tables S5 and S6.

		Census features				*OpenStreetMap*					
		Vertical density	Buildings age	Buildings age δ	Buildings	Roads inters.	POIs δ	POIs	$3^{rd} places\delta$	$3^{rd} places$	R^2
Age group	Less than 20	0.33866	0.04563	*0.00443*	0.58520	*0.02460*	*0.00178*	-0.12491	*0.01656*	*0.02024*	0.70
	20-29	0.30595	0.06459	*-0.00435*	0.56671	*0.03606*	*0.00835*	-0.15710	*-0.04202*	*0.06863*	0.62
	30-39	0.32186	0.04547	*0.00404*	0.58180	*0.02903*	*0.01305*	-0.18414	*-0.00932*	0.11118	0.69
	40-49	0.35208	0.04080	*-0.00558*	0.55456	*0.03222*	*0.01219*	-0.16717	*0.02372*	0.09274	0.72
	50-59	0.36537	0.05636	*-0.01636*	0.53545	*0.03144*	*0.01302*	-0.17297	*0.02237*	0.10675	0.72
	>=60	0.39468	0.03686	*-0.03155*	0.48057	*0.03235*	*0.02262*	-0.16020	*0.02306*	0.09441	0.69
City	Milano	0.00222	*0.00016*	*0.00000*	0.00359	*0.00006*	0.00029	-0.00090	*-0.00022*	*0.00025*	0.72
	Roma	0.00200	*-0.00038*	*-0.00041*	0.00208	*0.00019*	*0.00029*	*0.00008*	-0.00096	0.00090	0.52
	Palermo	0.00059	*0.00017*	*-0.00034*	0.00109	0.00260	*-0.00021*	*-0.00036*	0.00262	-0.00190	0.51
	Bari	0.00048	*0.00022*	*-0.00014*	*-0.00020*	*0.00015*	*-0.00007*	*0.00007*	*0.00012*	*0.00030*	0.14
	Venezia	0.00067	0.00035	*0.00002*	0.00090	*0.00051*	-0.00019	*0.00030*	0.00050	*-0.00055*	0.73
	Torino	0.00217	*0.00026*	*0.00011*	0.00442	*-0.00024*	*0.00005*	*-0.00071*	*-0.00014*	*0.00042*	0.82
	Napoli	0.00121	0.00108	*-0.00019*	0.00162	0.00141	*0.00019*	-0.00331	0.00188	0.00276	0.61
	**Aggregated**	0.36143	0.04634	*-0.01151*	0.55159	*0.03125*	*0.01334*	-0.16322	*0.01049*	0.08378	0.71

Table S5. See the caption of Table S4 for a description.

		Census features				*OpenStreetMap*					
	Age	Vertical density	Buildings age	Buildings age δ	Buildings	Roads inters.	POIs δ	POIs	$3^{rd} places\delta$	$3^{rd} places$	R^2
Milano	Less than 20	0.00036	*0.00003*	*0.00002*	0.00060	*-0.00001*	0.00004	*-0.00012*	*0.00000*	*0.00000*	0.72
	20-29	0.00019	*0.00001*	*0.00001*	0.00034	*0.00001*	0.00003	-0.00010	*-0.00003*	*0.00003*	0.70
	30-39	0.00030	*0.00002*	*0.00001*	0.00057	*-0.00001*	*0.00003*	-0.00014	-0.00006	*0.00006*	0.71
	40-49	0.00037	*0.00004*	*0.00001*	0.00062	*0.00000*	*0.00004*	-0.00015	*-0.00003*	*0.00004*	0.73
	50-59	0.00027	*0.00003*	*0.00000*	0.00045	*0.00001*	0.00003	-0.00011	*-0.00001*	*0.00004*	0.73
	>=60	0.00074	*0.00004*	*-0.00005*	0.00101	*0.00005*	0.00011	-0.00028	*-0.00008*	*0.00007*	0.69
Roma	Less than 20	0.00033	-0.00008	*-0.00006*	0.00037	*0.00002*	0.00005	*0.00000*	-0.00015	*0.00010*	0.52
	20-29	0.00017	*-0.00002*	*-0.00003*	0.00020	*0.00005*	*0.00004*	*-0.00002*	-0.00016	0.00014	0.33
	30-39	0.00024	*-0.00005*	*-0.00004*	0.00029	*0.00004*	*0.00004*	*0.00001*	-0.00017	0.00014	0.47
	40-49	0.00032	*-0.00007*	*-0.00007*	0.00036	*0.00003*	*0.00005*	*0.00001*	-0.00016	*0.00013*	0.52
	50-59	0.00028	*-0.00004*	*-0.00005*	0.00028	*0.00001*	*0.00004*	*0.00001*	-0.00011	0.00013	0.54
	>=60	0.00066	*-0.00013*	*-0.00015*	0.00060	*0.00004*	*0.00007*	*0.00002*	-0.00022	0.00032	0.51
Palermo	Less than 20	*0.00014*	*0.00003*	*-0.00007*	0.00028	0.00047	*-0.00005*	*0.00004*	0.00040	-0.00044	0.46
	20-29	0.00009	*0.00002*	*-0.00004*	0.00016	0.00025	*-0.00003*	*0.00003*	0.00026	-0.00024	0.50
	30-39	*0.00007*	*0.00002*	*-0.00004*	0.00018	0.00027	*-0.00002*	*0.00000*	0.00031	*-0.00023*	0.53
	40-49	*0.00008*	*0.00003*	*-0.00005*	0.00017	0.00040	*-0.00003*	*-0.00006*	0.00038	-0.00029	0.51
	50-59	0.00008	*0.00003*	*-0.00005*	0.00013	0.00035	*-0.00002*	*-0.00005*	0.00040	-0.00026	0.52
	>=60	*0.00011*	*0.00005*	*-0.00011*	0.00016	0.00091	*-0.00004*	*-0.00037*	0.00086	-0.00042	0.49

Table S6. See the caption of Table S4 for a description.

		Census features				*OpenStreetMap*					
	Age	Vertical density	Buildings age	Buildings age δ	Buildings	Roads inters.	POIs δ	POIs	$3^{rd} places \delta$	$3^{rd} places$	R^2
Bari	Less than 20	0.00010	*0.00004*	*-0.00003*	*-0.00004*	*0.00004*	*-0.00002*	*0.00002*	*0.00003*	*0.00005*	0.16
	20-29	0.00006	*0.00003*	*-0.00002*	*-0.00003*	*0.00002*	*-0.00001*	*0.00000*	*0.00002*	*0.00004*	0.14
	30-39	0.00007	*0.00003*	*-0.00002*	*-0.00003*	*0.00003*	*-0.00001*	*0.00002*	*0.00002*	*0.00003*	0.14
	40-49	0.00007	*0.00003*	*-0.00002*	*-0.00003*	*0.00002*	*-0.00001*	*0.00001*	*0.00002*	*0.00004*	0.15
	50-59	0.00006	*0.00003*	*-0.00002*	*-0.00002*	*0.00001*	*-0.00001*	*0.00001*	*0.00002*	*0.00005*	0.14
	>=60	0.00011	*0.00006*	*-0.00003*	*-0.00005*	*0.00002*	*-0.00001*	*0.00001*	*0.00002*	*0.00009*	0.11
Venezia	Less than 20	0.00011	0.00004	*-0.00001*	0.00017	*0.00006*	-0.00003	*0.00000*	0.00006	*-0.00004*	0.72
	20-29	0.00006	0.00003	*0.00000*	0.00008	0.00004	-0.00002	*0.00004*	0.00004	*-0.00006*	0.74
	30-39	0.00008	0.00004	*0.00000*	0.00012	*0.00005*	-0.00002	*-0.00003*	0.00006	*-0.00001*	0.70
	40-49	0.00011	0.00005	*0.00000*	0.00016	*0.00007*	-0.00003	*0.00004*	0.00007	*-0.00008*	0.73
	50-59	0.00009	0.00005	*0.00001*	0.00012	*0.00007*	-0.00003	*0.00010*	0.00007	*-0.00013*	0.72
	>=60	0.00023	0.00014	*0.00002*	0.00027	0.00024	-0.00006	*0.00013*	0.00018	*-0.00023*	0.72
Torino	Less than 20	0.00032	*0.00004*	*0.00001*	0.00073	*-0.00002*	*0.00001*	*-0.00008*	*-0.00002*	*0.00000*	0.83
	20-29	0.00019	0.00004	*0.00001*	0.00045	*-0.00003*	*0.00001*	*-0.00009*	*-0.00004*	*0.00006*	0.81
	30-39	0.00029	0.00007	*0.00003*	0.00066	*-0.00004*	*0.00001*	*-0.00020*	*-0.00002*	*0.00017*	0.81
	40-49	0.00034	*0.00005*	*0.00001*	0.0007	*-0.00003*	*0.00001*	*-0.00016*	*-0.00002*	*0.00011*	0.82
	50-59	0.00028	*0.00003*	*0.00001*	0.00057	*-0.00002*	*0.00000*	*-0.00010*	*-0.00002*	*0.00008*	0.82
	>=60	0.00074	*0.00001*	*0.00003*	0.0013	*-0.00011*	*0.00001*	*-0.00008*	*-0.00002*	*0.00000*	0.78
Napoli	Less than 20	0.00025	0.0002	*-0.00004*	0.00045	0.00033	*0.00004*	-0.00084	0.00041	0.00069	0.63
	20-29	0.00013	0.00013	*-0.00002*	0.00025	0.00019	*0.00002*	-0.00047	0.00025	0.00039	0.63
	30-39	0.00016	0.00014	*-0.00003*	0.00025	0.00020	*0.00002*	-0.00051	0.00029	0.00042	0.62
	40-49	0.00018	0.00016	*-0.00003*	0.00026	0.00020	*0.00003*	-0.00052	0.00030	0.00043	0.62
	50-59	0.00015	0.00014	*-0.00002*	0.00019	0.00018	*0.00002*	-0.00043	0.00024	0.00040	0.61
	>=60	0.00033	0.00032	*-0.00004*	0.00023	0.00031	*0.00005*	-0.00054	0.00038	*0.00044*	0.56

4 Univariate spatial models

In the *Main Text*, we presented the results of a series of multivariate spatial models which included all urban features in each model. For completeness, here we present the results of univariate models which include each urban feature separately. Note that we do not report the values of the pseudo R^2 since there would be a different value for each individual model, but the values are broadly consistent with those reported in the *Main Text.* Tables S7, S9 and S9 report the results of this analysis.

Table S7. Investigating the spatial relationship between urban features and vibrancy. We model the univariate relationship between the presence of people and urban features by constructing a series of spatial models to account for spatial correlations present in the data. The table shows the values of the model coefficients, and values are reported in italic if they are not significant after *fdr* correction ($p > 0.05$). Here, we present the results of these models when considering all cities together. Results for the individual cities are presented in Tables S8 and S8.

		Census features				*OpenStreetMap*				
		Vertical density	Buildings age	Buildings age δ	Buildings	Roads inters.	POIs δ	POIs	$3^{rd} places \delta$	$3^{rd} places$
Age group	Less than 18	0.21790	0.17622	-0.15166	0.23198	0.20835	*0.00655*	0.39968	0.39454	0.39739
	18-30	0.30393	0.23368	-0.18040	0.37554	0.24586	*-0.00808*	0.37994	0.54385	0.36934
	31-40	0.31049	0.22874	-0.18021	0.38101	0.24066	*-0.00611*	0.36311	0.51875	0.36098
	41-50	0.31160	0.22014	-0.17604	0.35909	0.24318	*0.00819*	0.36816	0.47865	0.36856
	51-60	0.31520	0.22024	-0.18868	0.34999	0.23390	*0.00363*	0.37846	0.49819	0.37832
	Over 60	0.34008	0.20211	-0.17702	0.36420	0.22572	*0.00907*	0.35644	0.47830	0.36211
City	Milano	0.18358	0.15319	-0.09980	0.21872	0.15975	*-0.01333*	0.37486	0.36460	0.37983
	Roma	0.29579	0.31141	-0.22084	0.32707	0.23774	*0.02782*	0.43080	0.57007	0.46366
	Palermo	0.32466	0.21122	-0.19182	0.28318	0.43345	*0.04981*	0.30940	0.70878	0.29113
	Bari	0.34266	*0.09057*	*-0.06386*	0.46819	0.50510	*0.10780*	0.36100	0.55429	0.42504
	Venezia	0.25368	0.13802	-0.18878	0.66212	0.52380	*0.01931*	0.39065	0.61652	0.38797
	Torino	0.34653	0.15354	-0.22997	0.29997	0.20917	*-0.01975*	0.40195	0.39820	0.30911
	Napoli	0.56667	0.40129	-0.24749	0.43056	0.66613	*0.02818*	0.41396	0.64335	0.38303
	**Aggregated**	0.28501	0.20859	-0.17398	0.32017	0.23281	*0.00409*	0.39391	0.47177	0.39238

Table S8. See the caption of Table S7 for a description.

	Age	Census features				*OpenStreetMap*				
		Vertical density	Buildings age	Buildings age δ	Buildings	Roads inters.	POIs δ	POIs	$3^{rd} places \delta$	$3^{rd} places$
Milano	Less than 18	0.11714	0.12296	-0.08116	0.13780	0.14343	*-0.00441*	0.37764	0.26656	0.38096
	18-30	0.22921	0.19125	-0.12339	0.28412	0.19631	-0.03492	0.39727	0.48550	0.40853
	31-40	0.24090	0.18963	-0.12304	0.29506	0.17305	-0.03654	0.37161	0.46876	0.37724
	41-50	0.21112	0.16016	-0.10671	0.25882	0.17476	*-0.01092*	0.37198	0.39689	0.37423
	51-60	0.21608	0.16206	-0.11157	0.25409	0.15838	*-0.01742*	0.35072	0.40867	0.35017
	Over 60	0.25690	0.16220	-0.09473	0.29268	0.13524	*-0.00746*	0.29912	0.40764	0.31160
Roma	Less than 18	0.24042	0.28740	-0.19453	0.27020	0.19588	*0.02168*	0.39981	0.52894	0.43396
	18-30	0.30824	0.35796	-0.26164	0.34383	0.26016	*0.01750*	0.49200	0.70421	0.47183
	31-40	0.31334	0.33898	-0.23415	0.37411	0.28792	*0.02908*	0.46596	0.64022	0.49272
	41-50	0.32268	0.31782	-0.22350	0.35615	0.25484	*0.03511*	0.43029	0.57119	0.47074
	51-60	0.31931	0.31829	-0.23015	0.35239	0.25298	*0.02927*	0.42924	0.57419	0.46737
	Over 60	0.37373	0.29327	-0.23676	0.38096	0.27073	0.04067	0.42126	0.50784	0.46343
Palermo	Less than 18	0.31445	0.20990	-0.20177	0.25757	0.43011	*0.05085*	0.30127	0.70697	0.28546
	18-30	0.32489	0.21262	-0.19049	0.33915	0.42506	*0.05013*	0.32869	0.70364	0.31129
	31-40	0.32226	0.21520	-0.17705	0.33754	0.43441	*0.04701*	0.31589	0.69757	0.29785
	41-50	0.32197	0.21067	-0.18413	0.27369	0.43544	*0.04981*	0.30260	0.69078	0.28348
	51-60	0.33058	0.21227	-0.18619	0.27983	0.43136	*0.05105*	0.31357	0.70942	0.29321
	Over 60	0.33206	0.19948	-0.18752	0.25185	0.42740	*0.04573*	0.29528	0.71453	0.27435

Table S9. See the caption of Table S7 for a description.

	Age	Census features				*OpenStreetMap*				
		Vertical density	Buildings age	Buildings age δ	Buildings	Roads inters.	POIs δ	POIs	$3^{rd} places\delta$	$3^{rd} places$
Bari	Less than 18	0.31478	*0.07048*	*-0.09411*	0.40755	0.47363	*0.10136*	0.35721	0.52676	0.43003
	18-30	0.32442	*0.09130*	*-0.04836*	0.49697	0.50747	*0.08141*	0.35413	0.55228	0.39682
	31-40	0.34528	*0.09162*	*-0.05173*	0.48555	0.50166	*0.09512*	0.32493	0.54842	0.38498
	41-50	0.37581	*0.11937*	*-0.02415*	0.53530	0.57132	0.14009	0.38198	0.59690	0.44681
	51-60	0.35888	*0.10239*	*-0.05420*	0.48560	0.49863	*0.11279*	0.35959	0.56426	0.42150
	Over 60	0.36996	*0.09966*	*-0.04831*	0.49212	0.50105	*0.11257*	0.36896	0.55664	0.42420
Venezia	Less than 18	0.24768	0.16184	-0.18293	0.67516	0.57224	*0.05674*	0.44810	0.56036	0.44461
	18-30	0.25865	0.11600	-0.18864	0.64168	0.47312	*-0.00975*	0.33493	0.63428	0.33524
	31-40	0.24934	0.10205	-0.19689	0.60514	0.43937	*-0.02356*	0.30616	0.63468	0.30333
	41-50	0.25530	0.12219	-0.19105	0.65430	0.50635	*0.00478*	0.37500	0.62843	0.37309
	51-60	0.25336	0.12732	-0.19510	0.64544	0.48451	*-0.00241*	0.35381	0.63248	0.34938
	Over 60	0.23569	0.12017	-0.16745	0.61690	0.46987	*-0.00163*	0.32952	0.63803	0.32803
Torino	Less than 18	0.25572	0.14353	-0.19245	0.17984	0.21934	*-0.00943*	0.36918	0.32257	0.28076
	18-30	0.39916	0.19159	-0.29318	0.36364	0.18527	-0.04635	0.46112	0.52041	0.35531
	31-40	0.38543	0.16394	-0.26779	0.37868	0.18795	*-0.04164*	0.40329	0.45712	0.30620
	41-50	0.38418	0.14882	-0.23628	0.36270	0.22508	*-0.01721*	0.40694	0.42004	0.31042
	51-60	0.38537	0.14696	-0.23558	0.34993	0.19527	*-0.01874*	0.40076	0.42331	0.31024
	Over 60	0.43116	0.14107	-0.22797	0.40199	0.18979	*-0.01469*	0.40535	0.39636	0.32351
Napoli	Less than 18	0.54796	0.39354	-0.26778	0.41343	0.66109	*0.02788*	0.46304	0.59493	0.43602
	18-30	0.56488	0.40599	-0.21917	0.45618	0.68495	*0.02991*	0.34363	0.67792	0.31162
	31-40	0.56721	0.39779	-0.23429	0.43959	0.67173	*0.02659*	0.37653	0.66100	0.34593
	41-50	0.57174	0.39949	-0.23470	0.43362	0.66671	*0.03190*	0.39154	0.65124	0.35824
	51-60	0.56942	0.40367	-0.25927	0.42372	0.64711	*0.02589*	0.44384	0.64649	0.41159
	Over 60	0.57460	0.39836	-0.23773	0.41297	0.63918	*0.02420*	0.39396	0.65529	0.36081
